# In-situ X-ray Differential Micro-tomography for Investigation of Water-weakening in Quasi-brittle Materials Subjected to Four-point Bending

**DOI:** 10.3390/ma13061405

**Published:** 2020-03-20

**Authors:** Petr Koudelka, Tomas Fila, Vaclav Rada, Petr Zlamal, Jan Sleichrt, Michal Vopalensky, Ivana Kumpova, Pavel Benes, Daniel Vavrik, Leona Vavro, Martin Vavro, Milos Drdacky, Daniel Kytyr

**Affiliations:** 1 Czech Academy of Sciences, Institute of Theoretical and Applied Mechanics, Prosecka 76, 190 00 Prague 9, Czech Republic; fila@itam.cas.cz (T.F.); rada@itam.cas.cz (V.R.); zlamal@itam.cas.cz (P.Z.); sleichrt@itam.cas.cz (J.S.); vopalensky@itam.cas.cz (M.V.); kumpova@itam.cas.cz (I.K.); benes@itam.cas.cz (P.B.); vavrik@itam.cas.cz (D.V.); drdacky@itam.cas.cz (M.D.); kytyr@itam.cas.cz (D.K.); 2 Czech Academy of Sciences, Institute of Geonics, Studentska 1768, 708 00 Ostrava-Poruba, Czech Republic; leona.vavro@ugn.cas.cz (L.V.); martin.vavro@ugn.cas.cz (M.V.)

**Keywords:** fracture propagation, in-situ X-ray 4D micro-CT, differential tomography, digital volume correlation, quasi-brittle materials, water-weakening, four-point bending

## Abstract

Several methods, including X-ray radiography, have been developed for the investigation of the characteristics of water-saturated quasi-brittle materials. Here, the water content is one of the most important factors influencing their strength and fracture properties, in particular, as regards to porous building materials. However, the research concentrated on the three-dimensional fracture propagation characteristics is still significantly limited due to the problems encountered with the instrumentation requirements and the size effect. In this paper, we study the influence of the water content in a natural quasi-brittle material on its mechanical characteristics and fracture development during in-situ four-point bending by employing high-resolution X-ray differential micro-tomography. The cylindrical samples with a chevron notch were loaded using an in-house designed four-point bending loading device with the vertical orientation of the sample. The in-house designed modular micro-CT scanner was used for the visualisation of the specimen’s behaviour during the loading experiments. Several tomographic scans were performed throughout the force-displacement diagrams of the samples. The reconstructed 3D images were processed using an in-house developed differential tomography and digital volume correlation algorithms. The apparent reduction in the ultimate strength was observed due to the moisture content. The crack growth process in the water-saturated specimens was identified to be different in comparison with the dry specimens.

## 1. Introduction

Fracture propagation characteristics in quasi-brittle materials induced by mechanical loading of the material have been intensively studied for many decades as the thorough understanding of all the related phenomena is essential in many fields including geology and civil engineering. Here, some of the most important characteristics are the conditions for the formation of the macroscopic crack and the mechanisms of its propagation. Among the established methods, particularly the approach based on the determination of the parameters of the nonlinear zone of material failure at the crack tip and its neighbourhood, the fracture process zone (FPZ), has attracted attention and has already been employed by Vesely and Frantik [1]. Experimental techniques based on various physical phenomena including the acoustic emission measurement and thermography have been developed by Vesely et al. [2]. However, since the FPZ is essentially a non-linear three-dimensional region, it is imperative that a method capable of assessing the volumetric FPZ characteristics has to be employed to ensure the sufficient reliability and repeatability of the results. We have already shown in a paper by Vavro et al. that a time-lapse high-resolution micro-tomography under three-point bending load conditions (a 4D micro-CT) provides sufficient insight into the crack formation process and the FPZ evolution mechanisms [3]. Additionally, we have demonstrated that the modification of the standard flexural arrangements into the four-point bending of a vertically oriented specimen brings significant advantages of the in-situ experiments in terms of uniform X-ray attenuation during tomographic scanning, improved resolution around the stress-concentrators, and the possibility to enhance the loading device mechanically, so that it is possible to perform intermittent loading with an arbitrary number of tomographic scans throughout the force-displacement (P-d) curve of the material. This involved the in-house development of a loading device with a vertical orientation of the investigated specimen, whose rotational axis is nearly identical with the rotational axis of the CT [4,5]. Thus, the optimal shape and dimensions of the cross-section with respect to the representative volume element of the investigated material can be selected and the length of the samples can be simultaneously increased without the need of increasing the diameter of the device. Here, the diameter increase always leads to a lower achievable resolution in the reconstructed 3D images and problems with the strong dependence of the X-ray attenuation on the projection angle.

It is well known that upon wetting, rocks show a reduction in the quality of their mechanical properties such as the compressive strength. This phenomenon was already mentioned by Hirschwald who first introduced the so-called softening coefficient to describe the loss of strength of test specimens after storing them in water [6]. The softening degree varies with different rock lithotypes. For crystalline rocks such as granites, the strength reduction is of a very low level, but porous sedimentary rocks, e.g., sandstones, are generally more sensitive to this effect.

A number of papers have been published on the influence of the moisture content on the decrease in the compressive strength of sandstones. According to, e.g., Morales Demarco et al. [7], who tested eight types of sandstones from different German localities, the specific values of the softening coefficient range from ca 0.5 to 1.0 for the individual sandstones. Similar findings of wide-ranging sandstone softening coefficient values were stated, for example, by Dyke and Dobereiner [8], Hawkins and McConnel [9] or Siedel [10]. The main factors determining the sensitivity of the sedimentary rock’s strength to the water content are, firstly, the pore radii distribution and, secondly, the amount and mineralogy of the matrix as well as the rock cement [7]. Therefore, the loss in compressive strength is higher for matrix-rich, clay-bearing sandstones in comparison with, for example, quartz-rich sandstones with a grain-supported texture [9,10]. It is also important to note that the decrease in strength of sandstone can be remarkable even when the rock is yet not fully saturated with water [7,11], in several cases with only 1% moisture content [12]. The lower strength due to the wetting is attributed to the reversible physicochemical interactions with the medium such as the physical adsorption, which leads to a decrease in the surface free energy of the solid; this is referred to as the “Rehbinder effect” [13].

Moisture contained in the rock also affects other material parameters than just the compressive strength. In this regard, the tensile strength is probably the rock property, which is far more sensitive to the effect of moisture than the compressive strength. According to Burstein [14], who tested two types of coal measures sandstones from the Sangar deposit in Russia, at the level of 1.5% moisture content, the tensile strength is reduced to one-third of that of the dry rock and at 6.5% moisture content, it becomes zero. The reduction in the tensile strength, up to 50% of the dry sandstone strength, has also been reported, for example, by Ojo and Brook and Ruedrich et al. [13,15]. In addition, as shown by Ruedrich et al. [15], there is a positive correlation between the decrease in the tensile strength and the intensity of the moisture expansion of sandstone. Similar findings on the role of the volume increase due to soaking have been reported for compressive strength by Morales Demarco et al. [7].

An effect equivalent to the decrease of the compressive strength with an increased moisture content of sandstones was observed also for the Young’s modulus [9,16]. A decrease in the Young’s modulus of water-saturated sandstones ranging between ca 20% and 50% against the dry state is reported by most authors [17,18,19].

The moisture content of sandstones also significantly reduces the fracture toughness, as was already demonstrated by Singh and Sun [20]. Although it is obvious that the rate of decrease in the fracture toughness due to moisture generally depends on the mineralogy and the grain size of sandstone, as well as the used testing method and specimen geometry [20], many authors stated an approximately 20% to 50% reduction in the fracture toughness related to the water saturation [17,21,22,23,24]. Additionally, the data about the dropping in the fracture toughness to approximately one-half to one-third of its value for dry sandstone can also be found in literature [20,25,26].

However, the evaluation of the water-weakening was, in these resources, based on either the mechanical properties or fracture characteristics without the ability to assess the response of the materials at the microstructural level on a volumetric basis.

In general, a high-resolution X-ray micro-CT represents a powerful non-destructive 3D imaging and analysis technique for studying the composition, internal texture and the behaviour of a wide range of objects and materials. This method was developed in the early 1970s and quickly became an indispensable part of medicine [27]. Subsequently, during the next two decades, the successful expansion of micro-CT into other fields of research such as wood science and technology, soil and marine science or palaeontology occurred very rapidly as demonstrated by Cnudde and Boone [28].

This method has recently become common in the Earth sciences and is attracting an increasing interest in this field, representing an effective tool particularly in the research of geomaterials (i.e., rocks and soils) or construction materials based on geomaterials such as building ceramics, concrete, mortars or renders. Within these materials, the microtexture features such as the total porosity, pore space geometry, pore-size distribution, cement distribution, grain size variation, density changes, natural fractures, hydraulic properties and fluid transport phenomena, as well as the failure behaviour belong to the most frequently studied phenomenon [28,29,30].

Geomaterials, and especially natural stones, were used for thousands of years in the construction of buildings and stone artworks, many of which are recognised as historically and culturally important heritage sites. In this context, X-ray micro-CT is currently widely applied for the 3D rock porosity assessment in relation to weathering resistance [31], often in mutual combination with mercury intrusion porosimetry [32,33,34]. The durability properties of natural stones are also negatively affected by the presence of the non-stable mineral phases, which oxidise easily, and a micro-CT can be successfully used to measure their content and volume distribution in some cases [32,35]. The assessment of water permeation properties of rocks is another example of the fruitful utilisation of the X-ray CT technique [36,37]. The migration of water and solutes throughout the rock pore space is generally considered to be one of the main reasons for the natural stone decay. In this research area, 4D X-ray micro-CT can be used, for example, to verify the inhibitory and preservation effects of hydrophobic agents reducing the permeability of rock surfaces and/or to study the dynamics of the crystallisation of harmful salts in the pores of geomaterials [38,39,40,41].

Recently, a wide range of applications is emerging for 4D X-ray micro-CT in the study of deformation behaviour and fracture processes in rocks. Crack visualisation, observation of the crack development and propagation, fracture process zone path length evaluation, crack tip opening displacement value estimation and local fracture toughness calculation may be mentioned as examples of these applications [4,5,42]. Furthermore, Skarżyński and Tejchman published the results on the combination of a three-point bending test and a subsequent high-resolution micro-CT imaging coupled with a structural analysis of a quasi-brittle building material [43].

In this paper, we use the advantages of novel in-situ flexural testing methodology based on an in-house developed instrumentation consisting of in-situ four-point bending setup with the vertical orientation of the sample and an X-ray CT scanner to assess the water-weakening effects in a natural rock. Mšené sandstone was selected as the material for the analysis thanks to its apparent response to the water content during the mechanical bending and its grain structure that can be successfully captured during deformation using the in-situ X-ray micro-CT. We study the water-weakening of the samples by identification of the changes in the acquired force-displacement diagrams and using advanced data processing based on differential tomography to assess the fracture characteristics and microstructural properties under the stress concentrator (notch). For this reason, the comparison of the deformation response of the dry and water-saturated samples in terms of the crack length and volume assessment, qualitative characteristics of fracture topology and force-displacement diagrams was performed.

## 2. Materials and Methods

### 2.1. Basic Characterisation of Mšené Sandstone

Mšené sandstone, quarried approximately 40 km NW from Prague, is a fine-grained, greyish white to light grey psammitic rock with a typical yellowish, ochre to brownish marbling. It is almost entirely (often more than 90 vol.%) composed of subangular to angular quartz grains. Other minerals such as feldspars, muscovite, tourmaline, zircon or titanite rarely occur, some of them even only as accessories. The average grain size of the quartz clasts ranges from 0.1 to 0.2 mm, the scarce flakes of muscovite are larger and reach up to 0.5 mm. The rock matrix (about 5 vol.%) is formed of kaolinite and is concentrated predominantly along the grain contacts or occasionally fills the inter-granular pores. The degree of secondary silicification is really low, authigenic silica overgrowths around the quartz grain rims can be observed only to a limited extent. The rock has a well-developed open unimodal pore system with an average pore size of about 30 μm [44,45].

**Figure 1:**
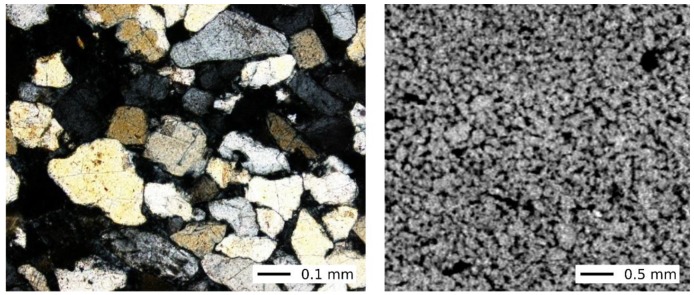
The microtexture of the Mšené sandstone—cross polarizers (XPL) optical microscopy in transmitted light (left), X-ray micro-CT of a dry sample (right).

It follows from the above that the quartz-rich Mšené sandstone is characterised by a weak rock compaction (diagenesis), of which the low content of the rock matrix, the long contacts prevailing between the quartz grains (see Figure 1) and the very low degree of secondary silicification are the microscopic indicators. The mentioned internal rock texture features are then reflected in the values of the basic material properties of the Mšené sandstone, for example, in its high porosity and water absorption capacity and, on the contrary, in its relatively low strength (see Table 1). Porosity is one of the basic petrophysical properties that most negatively affect rock strength. From this point of view, the rock under study represents the sandstone with the highest porosity among all sandstones currently quarried in the Czech Republic. The effective porosity of studied Mšené sandstone sample determined using AUTOPORE 9500 mercury porosimetry analyser (Micromeritics Instrument Corporation, Norcross, GA, USA) was 28.2%. This value is fully consistent with the data published for this sandstone by, e.g., Desarnaud et al. [40].

In our unpublished study, we have performed uni-axial compressive tests of the dry and water-saturated Mšené sandstone. The results showed that the average compressive strength decreased from 17.5 MPa for the dry rock samples to 16 MPa for the sandstone in a water-saturated state. The softening coefficient (i.e., the ratio between the compressive strength in the water-saturated and dry state) is, thus, 0.91 for the tested Mšené sandstone. This result is in good agreement with the previous works of Rybařík [46,47], where values in the range of 0.83–0.97 were reported. 

### 2.2. Specimens

**Figure 2:**
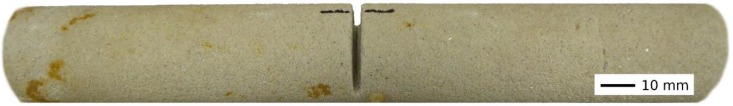
The ruptured specimen after the in-situ loading.

The specimens were prepared by drilling in a direction parallel to the sandstone bedding planes with a drilling speed of 63 rpm. Using a water-cooled diamond core drill bit with an outer diameter of 35 mm, cylindrical samples with a diameter of 28 ± 0.4 mm were prepared. Finally, the samples were cut to the length of 195.0 ± 2.0 mm. In the central part of the sample, a chevron notch was prepared using a circular diamond blade. Notch width represents one of the basic geometric parameters of used chevron bend specimens. According to the International Society for Rock Mechanics (ISRM) suggested method considered in this work [48], the notch width must be in a precisely defined relation to the specimen diameter, since it has the potential to significantly influence the observed material fracture toughness and fracture formation processes. Therefore, the notches were carefully cut resulting in the measured width in the range of 2.1–2.2 mm. The ruptured specimen is depicted in Figure 2.

To investigate the influence of the water content on the mechanical and fracture properties of the material, the selected samples were immersed in water until the full saturation of the material was reached. Before the flexural testing, a study to determine the water-saturation characteristics was performed. In this study, eight cylindrical samples of the Mšené sandstone were subjected to a long-term immersion in water and their weight was periodically inspected to assess the water saturation characteristics. The duration of the saturation experiment was 1128 h until the average relative weight-change between the two consecutive measurements was equal or less than 0.05%. During the saturation, the cylindrical samples were oriented vertically and the height of the water column was kept constant so that a 2 mm high part of the cylinders was above the water level and all the remaining faces of the specimens were in contact with the fluid. The controlled height of the water column was crucial in this study as the free surface exposed to the air guaranteed the capillary suction in the material microstructure. The study showed that 80% saturation was reached after approximately 24 h. However, the maximum saturation was, in all the cases, achieved after 7 weeks of immersion. For the purpose of this study, two dry specimens and two specimens immersed in water for 5 weeks reaching approximately 98% saturation were used.

### 2.3. Loading Device

**Figure 3:**
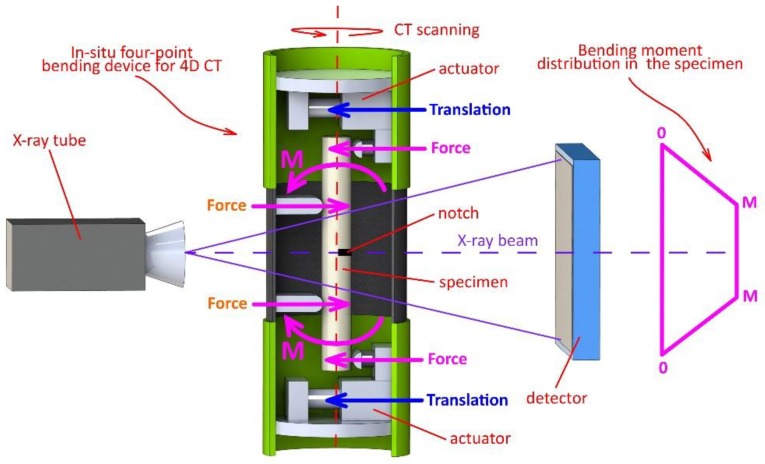
The principle of the in-situ experimental method.

**Figure 4:**
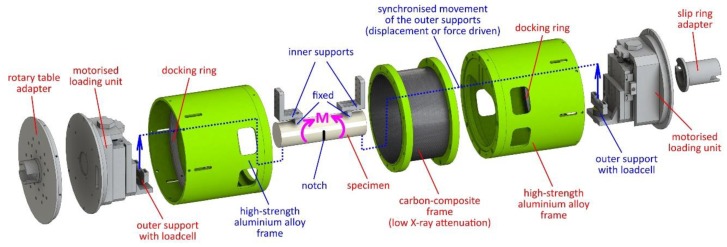
An exploded view of the device showing the individual components and their internal arrangement.

The in-house developed modular four-point bending device for in-situ experiments (Czech national patent 307897) was used for the in-situ experiments in this work. The detailed description of the device is provided in Kytyr et al. [4]. The modular design of the device is based on three main assemblies—two motorised loading units with actuators housing the supports of the four-point bending arrangement in a cylindrical load-bearing frame and a carbon fibre tube in-between the loading units. The central part of the device, where the notch is to be located on the specimens, is made of a carbon fibre composite tube with an MTM57 series epoxy resin matrix and T700S carbon fibres with a shell nominal thickness of 1.95 mm having low X-ray attenuation. The selected material ensures a high achievable contrast in the acquired radiograms. The central part is enclosed from both sides by two frames housing the driving units. The inner supports are in a fixed position during the loading, while the outer supports are movable and integrated with the driving units. The driving units accommodate precision captive stepper linear actuators (23-2210, Koco Motion DINGS, Morgan Hill, CA, USA), precision linear guideways (MGW12, Hiwin, Tokyo, Japan) and linear magnetic encoders with a resolution of 1 μm (LM10, Renishaw, Wotton-under-Edge, UK). The positions of the inner and the outer support can be independently adjusted to the optimal arrangement with respect to the geometry of the specimen and the nature of the investigated material. The force measurement is performed using the individual load cells (LCM300, Futek, Irvine, CA, USA) on both the outer supports. The maximum load capacity of the device is 1500 N per single support and the position accuracy and repeatability in a single-direction loading is better than 10 µm with a sensitivity better than 1 µm. Both the input and output wiring are equipped with a pair of slip rings that enables an infinite number of revolutions of the whole device during the tomographic scans. This feature easily allows for the experimental procedures to be performed with multiple revolutions during the tomographic scan of a single load step or for on-the-fly tomography with an uninterrupted loading. The control of the device is performed using an in-house developed closed-loop control system based on the LinuxCNC open-source software implemented on a real-time kernel described in detail by Rada et al. [49]. The specimen is mounted in the frame vertically and along the longitudinal axis of the device. The central part of the specimen with the notch is located within the carbon-composite tube. The principle of the in-situ experimental method is shown in Figure 3. An exploded view of the device showing the individual components and their internal arrangement is shown in Figure 4.

### 2.4. Loading Procedure

The outer supports were set to a span of 178.8 mm, while the inner supports were set to a span of 75.0 mm. After the settling of the specimen in the loading chamber on the outer supports, its stable position on the inner supports was achieved by applying the initial contact force of 5 N. Then, the inspection using the transmission radiography was performed to verify the correct position of the chevron notch tip and to exclude the samples with significant inhomogeneities in the volume of interest in the vicinity of the notch. 

The four-point bending loading sequence was performed as a combined displacement/force-driven experiment, where the movable supports form a master-slave couple. Here, one support acts as a master driven by the prescribed displacement and the other one follows as a force-driven slave using proportional-integral-derivative (PID) regulation. The loading rate of 60 μm/min was set for the master support. During the typical loading procedure, one load-step was performed during the material’s hardening phase, one load-step was performed at the ultimate-stress point and the remaining load-steps were performed during the post-peak softening phase. At each load step, a delay between the end of the loading and start of the tomographic scanning was used to minimise the influence of the mechanical relaxation.

### 2.5. Radiographical Imaging and CT Reconstruction

**Figure 5:**
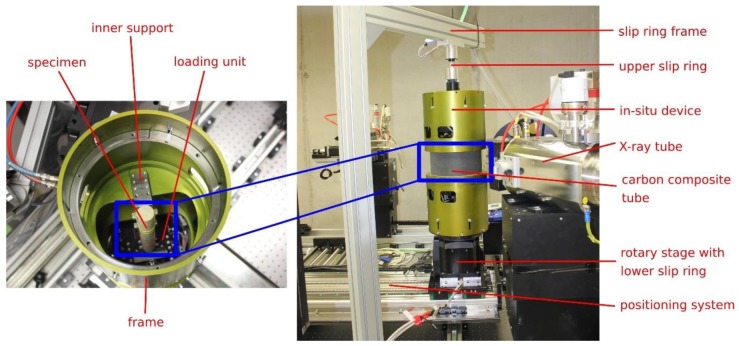
The in-situ loading device attached to the rotary stage of the X-ray scanner.

The time-lapse tomographic imaging of the bending experiments was performed using the TORATOM (Twinned Orthogonal Adjustable Tomograph) modular scanner. The scanner is composed of two perpendicular imaging lines with the capability to perform dual-source and dual-energy measurements. The geometry of both imaging lines is fully and independently adjustable thanks to a complex 13-axis positioning system. The scanner is equipped with two different X-ray tubes sharing part of their operational range to enable dual-source measurements. For imaging, different types of detectors are available, for instance flat panels with a Gd- or CsI-based scintillator or pixelated silicon detectors of the Medipix family. In this work, the measurements were performed using a combination of an XWT 240 SE microfocus reflection type X-ray tube (X-Ray WorX, Garbsen, Germany) with tungsten anode and a GOS scintillator flat-panel CMOS detector Dexela 1512 NDT (Perkin Elmer, Waltham, MA, USA). The detector is based on a 1944 × 1536 pixel matrix with a 74.8 μm pixel pitch and an active area of 145.4 mm × 114.9 mm. The setup geometry was set in order to achieve the best possible resolution in the region of interest comprising the chevron-edge notch. This resulted in a source-object distance of 120 mm and the source-detector distance of 530 mm leading to an approximately 4.42 geometrical magnification (reconstruction voxel size of approximately 17 μm). The X-ray tube was operated at an accelerating voltage of 180 kV and a target current of 206 μA leading to a target power of approximately 37 W. Transmission radiography was performed during every experiment to inspect the microstructural changes and crack formation during displacement increments as a supportive tool for selection of CT scans’ location. The tomographic scanning of the object was performed in 1200 angular positions with an angular step of 0.3 degrees and with a 500 ms exposure time leading to 18 min of scanning time per tomography. The CT reconstruction was performed in VG Studio Max (Volume Graphics, Heidelberg, Germany) using a filtered backprojection and cone-beam Feldkamp-Davis-Kress (FDK) algorithm. Due to the relatively high beam energy, a 2 mm Al filter was mounted in front of the X-ray source to limit the amount of the low-energy photons reaching the detector in the areas around the measured object. The presence of low-energy photons contributes to the saturation of the detector, but do not bear any new information, as they are not able to penetrate the object. The projections were acquired in an unsigned 16-bit binary format. They were all corrected using the standard flat-field method, which relies on an open-beam image (an image with the X-ray on and without the investigated object) and a dark-field image (an image with the X-ray off, in fact, an offset image of the detector). Both correction images were averaged from 1000 exposures for noise reduction. To avoid ring artefacts in the reconstruction, a bad-pixel correction was performed on the projections, approximating the damaged pixels with the averaged values from their neighbourhood. The in-situ loading device attached to the rotary stage of the X-ray scanner is shown in Figure 5.

### 2.6. Differential Tomography

In heterogeneous porous systems such as rocks, the changes in the microstructure induced by the mechanical loading such as the damage to the material, crack formation or propagation can only be visualized on the basis of tomographic reconstruction. In this work, the tomographic scans were performed at several load-steps throughout the force-displacement curve of the sample during the four-point bending experiment. As a result, the microstructural variations between two consecutive load steps are not only relatively small, but the four-point bending loading mode also causes the gradual translation and rotation of the material within the region of interest as the sample undergoes bending with the increasing displacement of the supports. Therefore, it is beneficial to employ the so-called differential tomography approach. Here, the 3D image from the current loading step is compared with the reference state (i.e., the 3D image of the undeformed specimen) to emphasize the microstructural variations and to suppress the static components. However, the two volumes have to be perfectly aligned for such an operation, otherwise the boundaries of the emphasised and the actual damage, related to the microstructural changes or fracture processes, can be even more hidden in the noise represented by the apparent blur in the 3D images. The aligning procedure has to be performed with a sub-voxel precision, but standard methods based on the global geometry of the object may not always be successfully employed as the boundaries of the sandstone samples are not well defined.

Let us denote the volume of the first unloaded state as the reference state and the volumes of the consecutive load steps as the loaded states. Then, the differential tomography procedure of the time-lapse experiment is composed of two measures—the precise alignment of the loaded states to the reference state and the subtraction of the reference state from each loaded state. A series of the loaded state volume differences is created, where the original structure is significantly suppressed and the analysis of the fracture processes is made possible. 

To achieve the precise aligning of all the volumes, a rigid transformation (i.e., the transformation considering translations and rotations only) is sought for each loaded state with respect to the reference state. Rigid transformation is used to avoid the deformation of the transformed volume. The transformation is given by seven parameters: three parameters $p_{1},\;p_{2},\;p_{3}$ defining the axis of rotation, Θ defining the angle of rotation and three parameters $t_{1},t_{2},t_{3}$ defining the translations. The matrix R of the rotational part of the transformation in three dimensions can be written as

$$R\left(p_{1},\;p_{2},\;p_{3},\;\Theta \right)=\left(1-\cos\Theta \right)A+\cos{\Theta I}_{3}+\sin\Theta \;C,$$

where

$$A=\left[\begin{array}{ccc} p_{1}^{2} & p_{1}p_{2} & p_{1}p_{3}\\ p_{1}p_{2} & p_{2}^{2} & p_{2}p_{3}\\ p_{1}p_{3} & p_{2}p_{3} & p_{3}^{2} \end{array}\right],$$

$$I_{3}=\left[\begin{array}{ccc} 1 & 0 & 0\\ 0 & 1 & 0\\ 0 & 0 & 1 \end{array}\right],$$

$$C=\left[\begin{array}{ccc} 0 & -p_{3} & p_{2}\\ p_{3} & 0 & -p_{1}\;\\ -p_{2} & p_{1} & 0 \end{array}\right].$$

The axis of rotation has to be a unit vector satisfying the condition

$$\sqrt{p_{1}^{2}+p_{2}^{2}+p_{3}^{2}}=1,$$

Together with the parameters of the translation, the transformation matrix has the form

$$T\left(p_{1},\;p_{2},\;p_{3},\;\Theta ,t_{1},t_{2},t_{3}\right)=\left[R\;\;\begin{array}{c} t_{1}\\ t_{2}\\ t_{3} \end{array}\right],$$

where R is an orthogonal transformation (i.e., $R^{T}=R^{-1}$). In addition, det(R)=1 so that R does not produce a reflection. Hence, it represents a rotation as an orientation-preserving orthogonal transformation. The parameters of the transformation are determined by selecting small sub-volumes in the reference state and then by obtaining their positions in the loaded states using the digital image correlation (DIC) algorithm. Let us denote the sub-volumes as the regions of interest (ROIs). The centroids of the ROIs in the reference state are selected preferably outside the expected region of deformation to facilitate finding the corresponding positions of the ROIs in the loaded states. From our experience with the sandstone samples subjected to four-point bending, three ROIs not located on one line are usually enough for determining the transformation, but a higher number is preferable in order to improve the accuracy. In the case of this work, eight ROIs were used. For the sub-voxel accuracy, the voxel with the highest value of the correlation coefficient is selected together with the additional subset of all the neighbouring voxels (the voxels surrounding the voxel with the highest value of the correlation coefficient even in the corners, i.e., considering the 26-connected type of connectivity). These 27 points are then interpolated by a second-order polynomial. The maximum of this polynomial is determined and the corresponding coordinate gives the sub-voxel displacement. The ROI(s) with low correlation coefficients are omitted from the transformation search process to improve the accuracy. The described procedure is robust and accurate although it is relatively computationally expensive.

The parameters of the rigid transformation are calculated by the minimisation of the Euclidian distance between the ROI coordinates in the reference state and the corresponding positions of these ROIs in the loaded state. For each loaded state, the function

$$\underset{i}{\sum }||T\left(p_{1},\;p_{2},\;p_{3},\;\Theta ,t_{1},t_{2},t_{3}\right)\left[\begin{array}{c} \tilde{x_{i}}\\ 1 \end{array}\right]-x_{i}||{}_{2}$$

where $x_{i}$ is the position of the centre of the ROI in the reference state, $\tilde{x_{i}}$ is the corresponding ROI centre position in the loaded state and the $||a_{2}||=\sqrt{\underset{i}{\sum }a_{i}^{2}}$ Euclidean norm is minimised. The minimisation is performed by the application of the global minimisation function from the MATLAB (Mathworks, Natick, MA, USA) database. 

## 3. Results

### 3.1. Force-Displacement Diagram

**Figure 6:**
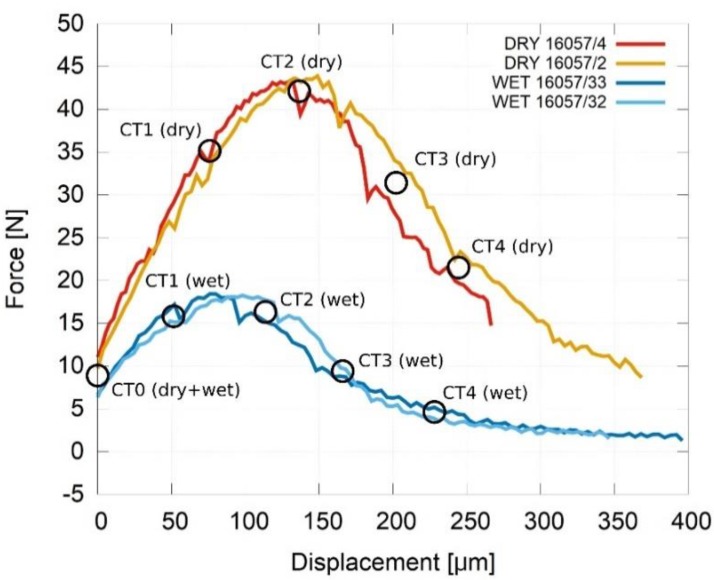
The force-displacement diagram showing the difference between the dry and the wet specimens and approximate locations of the CT scans.

The force-displacement diagram was evaluated using the force and displacement recorded during the experiments by the in-situ device control system. As the position indicated by the linear encoders was used for the evaluation of the displacement, the influence of the machine stiffness and its elastic deformation was partially corrected during the data processing. The force-displacement diagram showing the difference between the dry and wet specimens is presented in Figure 6. The approximate locations of the CT scans are also highlighted in the graphs. The behaviour of the dry and the wet specimens was, in all the cases, significantly different in terms of the maximum force. The maximum force of the wet specimens was lower by approximately 60% in comparison with the dry specimens (dry approximately 45 N, wet approximately 18 N). The inspection using the transmission radiography performed between the load-steps concentrated on the notch showed that the major microstructural changes (e.g., crack initiation) appeared at the approximately identical points of the loading curve relative to the peak force displacement of the given sample. The decreases in the macroscopic force-displacement diagrams are caused primarily by two sources. In the displacements between the individual CT scans, the repeatedly occurring force-decreases can be attributed to settling of the material near the contact with the supports, where the relatively low contact area induces release of individual grains from the binder. In the vicinity of the CT scans and at higher loads, the decreases are connected with the relaxation before or during the tomographical scans. This can be eliminated using “on-the-fly” scanning mode, but in this case, it was technically impossible due to the unsuitable displacement/CT-scanning time ratio.

### 3.2. CT Reconstruction

**Figure 7:**
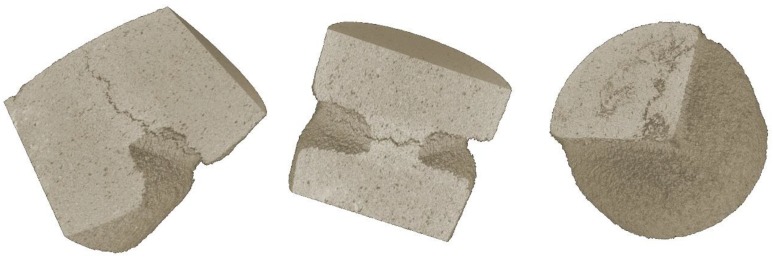
The visualisation of the crack in the last damage state (CT4) of a wet specimen.

The parameters of the CT scanning procedure ensured that the quality of the reconstructed 3D images was sufficient for the further processing using the differential tomography analysis. The visualisation of the typical reconstructed volume showing the area of the notch and the propagating crack is shown in Figure 7. This particular example represents the crack in the wet specimen in the last loading step. It can be seen that the morphological features of the sandstone, the macroscopic crack including the tip and the microstructural changes in the vicinity of the macroscopic crack are captured with high quality.

### 3.3. Differential Tomography

**Figure 8:**
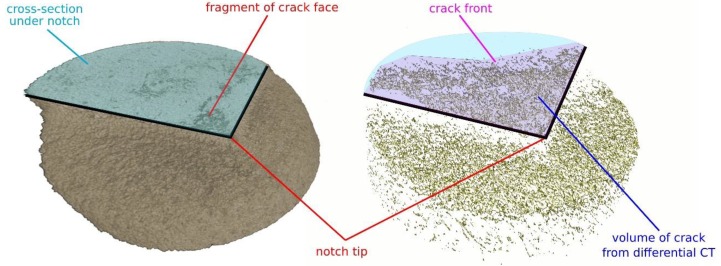
The schematics of the tomographic visualisation in the region of the notch (CT4 (wet) loading state, left), the 3D difference between the CT4 (wet) and CT0 (wet) (right).

**Figure 9:**
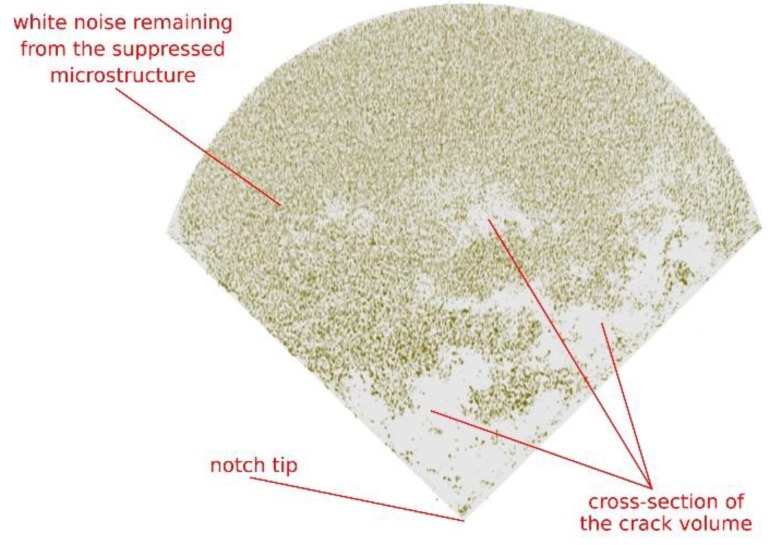
Cross-section of the differential tomography between the CT3 (wet) and CT0 (wet), smoothed by the floating average from eleven neighbouring slices. The crack is emphasized (grey regions), while the stone microstructure practically disappeared—only white noise remained.

Using the differential tomography algorithm described in Section 2.6, it was possible to precisely align the reconstructed volumes and to highlight the macroscopic crack formation and its propagation in the 3D images. The 3D difference of the last loading step and the reference scan of the wet specimen are depicted in Figure 8 showing the same specimen with the loading-step as provided in Figure 7. In the left part of the figure, the region of interest around the notch captured in the CT4 (wet) loading state of the wet sample is depicted together with the graphically highlighted stress-concentrator geometry. Together with the 3D visualization of the material microstructure, the fragments of the crack-face are visible as darker regions in the cross-section of the chevron notch. In the right part of the figure, the output from the differential CT of the same region of interest is shown. Aside from the identified changes in the microstructure, which are visible as a speckle pattern within the circumference of the specimen shape, the difference of the two loading states reveals the crack topology, which remains hidden in standard tomography. As a result, the difference between the crack fragments apparent in the standard CT and the actual crack is visible together with the shape of the crack front, which is one of the important fracture characteristics. Additionally, visualization of an average difference between the CT0 (wet) and CT3 (wet) load-steps calculated from eleven neighbouring slices of the notch-tip region-of-interest for the dry specimen is provided in Figure 9. This figure represents the planar visualisation of the data shown in the right part of Figure 8, when the intensities in the differential volume are smoothed by floating average in the direction parallel to the specimen’s longitudinal axis to demonstrate the crack topology identified by the differential tomography. 

**Figure 10:**
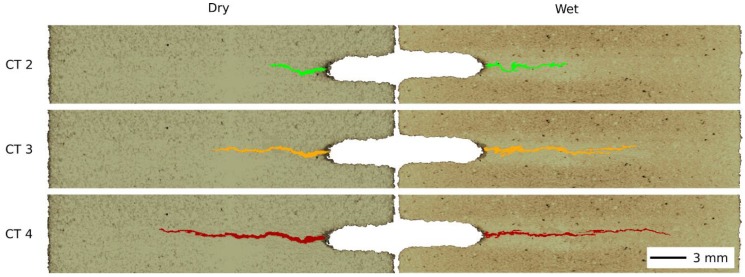
Comparison of the crack-shape based on the differential tomography for both dry and wet specimens in the post-peak softening phase.

The comparison of the slices in the reconstructed 3D images processed using the differential tomography for both the dry and the wet specimens with the highlighted identified cracks is shown in Figure 10. The comparison is performed at similar points in the force-displacement curves in the post-peak softening phase after the formation of the macro-crack (compare the position of the load steps provided in Figure 6) to show the difference in the crack shape and length. In every case, the images show the superposition of the identified crack volume over the reconstructed 3D image in the plane under the notch tip. The direct comparison between the dry and water-saturated specimen shows apparently different geometrical characteristics of the cracks as well as differences in the crack topology. Generally, the flexural loading of the wet specimens leads to a higher crack length and lower crack volume, which is consistent with the acquired force-displacement diagrams. The topological difference primarily consists in the tendency of the wet specimen to kink in terms of the higher number of kinks, the higher kink angles and lower bend radii. Furthermore, the detailed inspection of the wet-crack topology reveals that branching of the cracks that are not present in the dry specimens can be observed. Table 2 summarizes the influence of the wetting on the measured crack length and the calculated crack volume.

## 4. Discussion

In this study, a novel in-house developed four-point bending loading device with a vertical orientation of the sample was successfully employed for the in-situ four-point flexural testing of dry and water-saturated specimens of Mšené sandstone in an X-ray micro-CT scanner. The reconstructed 3D images were processed using an in-house differential tomography algorithm based on the image transformation and the DIC technique in order to evaluate the differences in the volumes and to investigate the crack initiation and propagation phenomena. It was proven that significant differences in the deformation behaviour can be identified between the dry and wet specimens of the investigated material. The main results and findings can be summarised in the following points:

- The utilised processing of the tomographic data enabled to precisely align the reconstructed 3D images and consequently perform the differential tomography procedure in order to reliably highlight the changes in specimen microstructure that occurred between the load steps. Here, the notch opening and deformation of the specimen render the common 3D image registration techniques and the subtraction of the volumes unsuitable for this application. As a result of the sub-voxel deformation of the specimen including its bending, minor movement and notch opening, the real crack shape and propagation would remain hidden in the noise.
- The force-displacement diagram was measured using the in-situ loading device and a significant difference between the dry and water-saturated specimen was identified in terms of the mechanical response. The maximum force reached in the experiments with the wet specimens was approximately 60% lower and occurred at approximately 50% lower displacement than during the experiments with the dry specimens. The resulting overall shape of the force-displacement diagrams was similar for both the dry and water-saturated specimens, but the slope of both the hardening and post-peak softening phase was lower in the case of the wet specimen.
- The mechanical behaviour of the investigated material and related water-weakening effects were in good agreement with the existing literature, where the observed peak force reduction by 60% for the water-saturated material was in agreement with results published by e.g., Singh and Sun, Nara et al. or Seto et al. [20,25,26]. However, the substantial contribution of the conducted research consists in the possibility to describe both qualitatively and quantitatively the differences in the crack geometrical properties and topology together with the corresponding microstructural response of the material and to perform a direct comparison between the dry and the water-saturated specimens. To the best of our knowledge, similar experiments have not been performed on natural quasi-brittle materials yet.
- It was possible to identify the differences in the crack characteristics of the dry and the water-saturated specimens using the differential tomography. In the results section, we have shown, for instance, the evolution of the crack length and the crack volume in the post-peak softening phase of the material response. The results revealed that the saturation of the specimens leads to higher crack length, while the crack volume is lower than in the dry state. Moreover, as the 3D image of the notch region is the output of the differential tomography procedure, other geometrical characteristics including the crack can be estimated. Furthermore, we have commented on the qualitative evaluation of the crack topology, where apparent differences in the kinking and branching of the crack were observed. Additionally, an arbitrary quantitative crack evaluation including fractal dimension calculations can be performed using the 3D image of the identified crack volume produced by the differential tomography algorithm.

## 5. Conclusions

The in-situ X-ray micro-CT flexural testing of a natural quasi-brittle building material in dry and water-saturated state was performed using an in-house developed instrumentation and an in-house developed data processing methodology. Two sets of cylindrical specimens with a chevron notch were prepared for the experimental study: dry specimens and water-saturated specimens subjected to long-term immersion in water resulting in approximately 98% saturation. During the time-lapse micro-CT under loading, several load-increments throughout the force-displacement were established to capture the deforming microstructure of the specimens. The reconstructed 3D images were subjected to a differential tomography procedure, where image transformation and DIC techniques were used to precisely align the acquired volumes to enable the calculation of the microstructural differences of the samples. Hence, from all the measurements, the 3D image from every load-step was compared against the 3D image of the unloaded state to highlight the crack initiation and propagation process. It was found out that the use of a differential CT is necessary as the 3D image of every load-step only provided information related to the visible fragments of the crack faces. Using such a methodology, the mechanical and fracture characteristics connected with the water-weakening of the material were evaluated on the basis of the data from the loading device and the tomographical scanner. The water content in the material resulted in an approximately 60% decrease in the peak-force, which was achieved at approximately 50% lower displacement, while the slopes of the hardening and post-peak softening phase of the material response were also significantly lower. The crack characteristics were studied on a volumetric basis using the differential CT datasets and both the geometrical and topological differences between the dry and water-saturated material were identified. The crack lengths were calculated showing a higher overall length of cracks in the water-saturated material and an inversely proportional dependence of the crack length on the prescribed displacement. Simultaneously, the identified crack volumes followed the changes in the mechanical properties influenced at the microstructural level by the water content as the lower measured forces were complemented by lower volume of the cracks. The topology of the cracks was studied qualitatively from the 3D images of the cracks resulting in the significant kinking and branching of the crack observed in the water-saturated specimen, when the crack is analysed in detail in the region of the notch tip. Thus, the presented methodology forms the basis for additional research related to the weathering of the quasi-brittle building materials including the calculation the fractal dimension characteristics.

## Figures and Tables

**Table 1:** The basic physical and mechanical properties of the Mšené sandstone (adopted from Rybařík [45,46]).

**Bulk Density** **[kg/m^3^]**	**Total Porosity** **[% by Volume]**	**Water Absorption** **[% by Weight]**
1850–1930	26.3–29.7	10.8–13.3
**Compressive Strength (Dry)** **[MPa]**	**Compressive Strength** **(Water-Saturated)** **[MPa]**	**Flexural Strength (Dry)** **[MPa]**
23–33	19–32	0.9–1.6

**Table 2:** The crack length and the crack volume of the dry and wet specimen.

Load Step	Crack Length: Dry [mm]	Crack Length: Wet [mm]	Crack Volume: Dry [mm^3^]	Crack Volume: Wet [mm^3^]
CT 2	4.67	6.69	14.53	8.48
CT 3	9.33	12.44	51.52	27.18
CT 4	13.22	15.09	73.33	34.16
